# Role of Adipokines and Perivascular Adipose Tissue in Abdominal Aortic Aneurysm: A Systematic Review and Meta-Analysis of Animal and Human Observational Studies

**DOI:** 10.3389/fendo.2021.618434

**Published:** 2021-03-15

**Authors:** Shivshankar Thanigaimani, Jonathan Golledge

**Affiliations:** ^1^ The Queensland Research Centre for Peripheral Vascular Disease (QRC-PVD), College of Medicine and Dentistry, James Cook University, Townsville, QLD, Australia; ^2^ The Australian Institute of Tropical Health and Medicine, James Cook University, Townsville, QLD, Australia; ^3^ The Department of Vascular and Endovascular Surgery, Townsville University Hospital, Townsville, QLD, Australia

**Keywords:** adipokine, adipose tissue, periaortic adipose tissue, abdominal aortic aneurysm, aortic rupture

## Abstract

Improved understanding of abdominal aortic aneurysms (AAA) pathogenesis is required to identify treatment targets. This systematic review summarized evidence from animal studies and clinical research examining the role of adipokines and perivascular adipose tissue (PVAT) in AAA pathogenesis. Meta-analyses suggested that leptin (Standardized mean difference [SMD]: 0.50 [95% confidence interval (CI): −1.62, 2.61]) and adiponectin (SMD: −3.16 [95% CI: −7.59, 1.28]) upregulation did not significantly affect AAA severity within animal models. There were inconsistent findings and limited studies investigating the effect of resistin-like molecule-beta (RELMβ) and PVAT in animal models of AAA. Clinical studies suggested that circulating leptin (SMD: 0.32 [95% CI: 0.19, 0.45]) and resistin (SMD: 0.63 [95% CI 0.50, 0.76]) concentrations and PVAT to abdominal adipose tissue ratio (SMD: 0.56 [95% CI 0.33, 0.79]) were significantly greater in people diagnosed with AAA compared to controls. Serum adiponectin levels were not associated with AAA diagnosis (SMD: −0.62 [95% CI −1.76, 0.52]). One, eight, and one animal studies and two, two, and four human studies had low, moderate, and high risk-of-bias respectively. These findings suggest that AAA is associated with higher circulating concentrations of leptin and resistin and greater amounts of PVAT than controls but whether this plays a role in aneurysm pathogenesis is unclear.

## Introduction

Screening studies suggest that abdominal aortic aneurysms (AAA) prevalence is approximately 3% in men and 1% in women aged over 65 years (1). The main complication of AAA is aortic rupture which is estimated to cause 200,000 deaths worldwide each year (2). Currently the management of AAA is limited to surgical repair (3). Randomized clinical trials have demonstrated that early elective surgical repair does not reduce mortality for people with small asymptomatic AAAs (4, 5). Current guidelines therefore recommend simply monitoring small (<50mm in women and <55m in men) asymptomatic AAAs (6). Most small AAAs continue to expand and eventually reach a diameter when surgical treatment is indicated (6). Drug therapies which limit growth of small AAAs would be valuable in clinical practice. Previous small randomized controlled trials have however failed to identify any effective medications (7).

Adipocytes are fat depots that store excess energy as triglycerides. They also have an endocrine function by secreting adipokines, such as leptin, adiponectin, dipeptidyl peptidase-4 (DPP-4), resistin and resistin-like molecule beta (RELMβ) (8). Leptin is an adipokine involved in regulating energy homeostasis and obesity. It is considered an important regulator of β cell mass and survival (9). In patients with leptin deficiency, recombinant leptin replacement therapy suppresses appetite and increases energy expenditure and leptin has been developed as a treatment for obesity (10). The adipokine adiponectin has insulin-sensitizing, anti-inflammatory and anti-apoptotic effects (11). Adiponectin and agonists of its receptor have also been suggested as a treatment for obesity and its complications (12). DPP4 release has been shown to strongly correlate with adipocyte size, potentially representing an important source of DPP4 in obesity (13). Resistin belongs to an RELM family and is believed to be an important link between obesity, insulin resistance, and diabetes (14). Increasing evidence suggests that resistin plays an important role in a variety of biological processes involved in cardiovascular diseases, autoimmune disease, and asthma (14). RELM-β is another member of RELM family that is mainly secreted from the adipose tissue in rodents (15) and from adipose-associated macrophages in humans (16). Adipose is deposited over multiple subcutaneous and visceral sites but when considering a role in aortic pathology, perivascular aortic adipose tissue (PVAT) is of particular interest. Adipose tissue residing in the vascular adventitia has been proposed to act like endocrine cells that respond to inflammatory stimuli by releasing adipokines and other signaling mediators to maintain vascular homeostasis and potentially play a role in aortic pathology (17, 18). This review summarizes published animal and human studies that investigated the role of these adipokines and PVAT in AAA pathogenesis.

## Methods

### Identification and Inclusion of Studies in This Review

Studies were identified from the PubMed database published until 5^th^ August 2020 using the following search terms [“adiposity” OR “adipose tissue” OR “adipokines” OR “adiponectin” OR “leptin” OR “resistin” OR “subcutaneous fat” OR “visceral fat” OR “peri aortic fat” OR “obesity”] AND [“Abdominal aortic aneurysm” OR “aortic occlusive disease” OR “aortic rupture”]. Animal and observational human studies that investigated the role of adipokines or PVAT in AAA were included. Outcomes included aortic diameter and adipokine concentrations or relative amount. Included articles were identified by one author (ST) and reviewed by another author (JG). A minimum of two studies reporting the aortic diameter in animal studies and adipokines levels or PVAT in human studies were required for meta-analyses to be performed. In studies where the effect of deficiency and inhibition of a specific adipokine or PVAT were reported, data were converted to an equivalent format in order to include in the same meta-analyses. Meta-analysis findings were presented as standardized mean difference (SMD) and 95% confidence intervals (CI). Meta-analyses were performed using random effects model and an inverse variance method was used to estimate the heterogeneity between studies. All analyses were conducted using the “meta” package of R software version 3.4.4. p value of ≤0.05 were considered significant.

The quality of the animal studies was assessed using a modified version of the Animal Research: Reporting of In Vivo Experiments (ARRIVE) guideline criteria. The quality of the included human studies was assessed using the modified version of Newcastle-Ottawa Scale 10 which assessed the study design, sample size estimation, age and sex-matching of controls, whether imaging was performed to measure AAA size, method of AAA size assessment, adjustment for confounders, and whether the observers were blinded to group during analysis. Assessment scores with <50, 50–75, and >75% were considered to have high, moderate, and low risk of bias respectively.

## Results and Discussion

**Figure 1:**
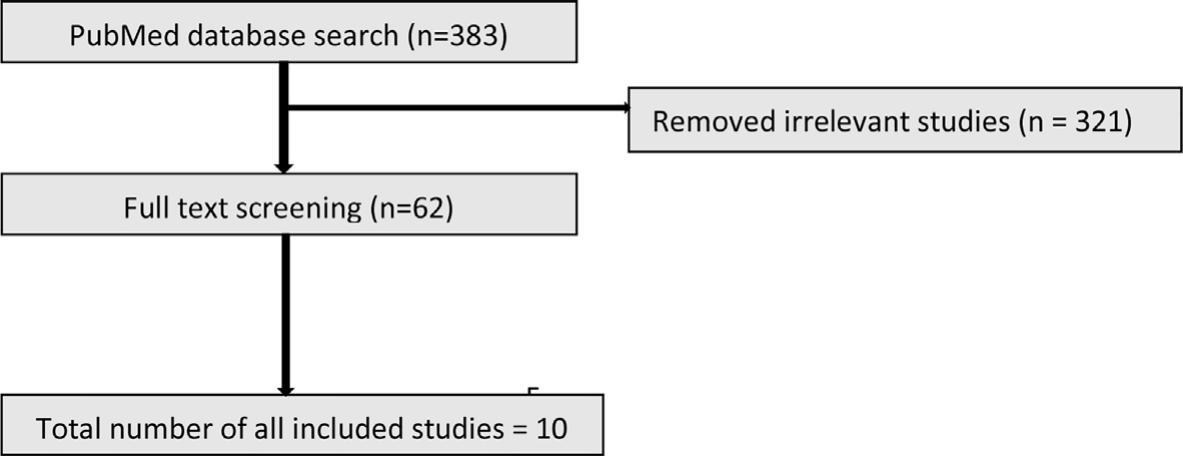
PRISMA flow diagram illustrating the process of selection of the animal studies.

**Figure 2:**
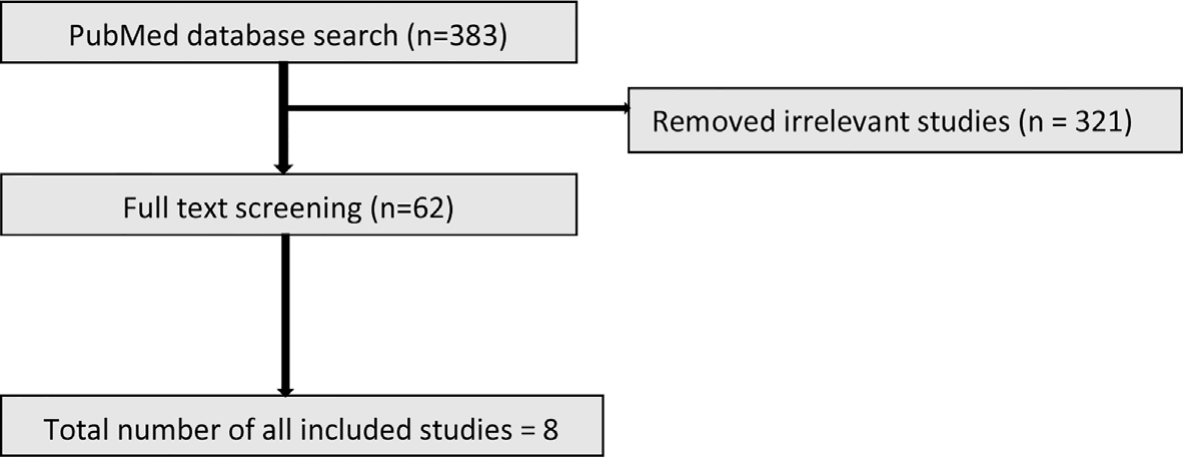
PRISMA flow diagram illustrating the process of selection of the human studies.

The initial search identified 383 studies. Based on the inclusion criteria, 321 studies were excluded due to not meeting the entry criteria. The full text of the remaining 62 studies (19 animal and 43 human) were reviewed of which ultimately 10 animal and 8 human studies were included (**Figures 1** and **2**).

### Animal Studies Investigating the Role of Adipokines or PVAT in AAA

Ten eligible rodent studies were identified (19–28). These studies included a total of 319 rodents in which the effects of leptin, resistin, adiponectin, and PVAT were investigated (19–28). Detailed information about each study is shown in **Table 1**.

**Table 1:** Animal studies investigating the role of adipokines and PVAT in AAA.

Ref	Animal strain	Intervention	AAA model	Groups	Sample size	Assessment period	Aortic diameter (mm)	Mechanisms implicated in AAA formation
*A) Animal studies investigating the effect of leptin on aortic diameter*								
(19)	C57BL/6J mice	Peri-aortic leptin application	Ang-II	Placebo	16	28 days	1.5 ± 0.1	Para-visceral aortic leptin in ApoE^–/–^ mice induces local medial degeneration and augments angiotensin II-induced AAA.
				Leptin + Ang-II	16		1.8 ± 0.2	
(21)	C57BL/6J mice	Germline deficiency in leptin	Ang-II	Control	10	28 days	NR	Leptin deficient obese mice exhibiting AAAs had greater macrophage content in visceral adipose tissue than mice not developing AAA.
				High-fat	25			
				LDLr−/−	7			
				Leptin−/− Ob+	10			
				Leptin−/− Ob/Ob	10			
(20)	C57BL/6J mice	Diet induced obesity	CaCl_2_	ApoE−/− no WAT control	9	28 days	0.1 ± 0.03	Perivascular implantation of adipose tissue from either diet induced obese mice or lean mice exacerbated AAA development, but this was abolished in leptin-deficient obese mice.
				ApoE−/− obese WAT	7		1.1 ± 0.5	
				ApoE−/− lean WAT	9		2.1 ± 0.9	
				ApoE−/− Ncc−/− IL18r−/− obese WAT	8		0.6 ± 0.3	
(22)	C57BL/6J mice	I.P. injection	Ang-II	Sham + Saline	8	28 days	1.0 ± 0.2	Pre-treatment with leptin significantly downregulated protein expression of the Th2 cytokine IL-4 and mRNA levels of GATA-3, the key transcription factor for Th2 polarization, and significantly upregulated Th1 cytokine INF-γ and T-bet, the key transcription factor for Th1 polarization
				Ang-II control	12		2.3 ± 0.6	
				Ang-II + leptin	12		1.5 ± 0.4	
*B) Animal studies investigating the effect of adiponectin on aortic diameter*								
(23)	ApoE−/−mice	Recombinant adenoviral vector encoding mouse adiponectin	Ang-II	PBS-infused control (LDLr−/−)	8	56 days	0.51 ± 0.05	APN inhibited the angiotensin type-1 receptor (AT1R), inflammatory cytokine and mast cell protease expression, and induced upregulation of LOX in the aortic wall, improved systemic cytokine profile and attenuated adipose inflammation, thus preventing AAA
				AdAPN mice (LDLr−/−)	8		0.53 ± 0.12	
				AdGFP mice (LDLr−/−)	12		0.88 ± 0.45	
(24)	C57BL/6J mice	Adiponectin gene deficiency	Ang-II	Control AAA	18	28 days	1.67 ± 0.09	Adiponectin-deficiency augmented the early infiltration of macrophages and increased the expression of pro-inflammatory factors in the dilated aortic wall, contributing to the elevated incidence of AAA
				APN−/− AAA	18		2.12 ± 0.07	
*C) Animal studies investigating the effect of Resistin-like molecule-beta on aortic diameter*								
(25)	ApoE−/− mice	Deletion of RELMβ	Ang-II	AAA only	15	28 days	1.0 ± 0.1	Deletion of RELMβ may reduce the expression of pro-inflammatory cytokines, MMP-2 and MMP-9 *via* ERK1/2 and JNK activation, resulting in AAA attenuation
				Sham	15		2.1 ± 0.5	
				Si-NC	15		2.1 ± 0.5	
				Si- RELMβ	15		1.4 ± 0.3	
(26)	ApoE−/− mice	RELMβ mRNA and protein levels	Ang-II	Control	NR	28 days	NR	Increased RELMβ mRNA and protein levels contribute to aneurysm formation
				AAA	NR			
*D) Animal studies investigating the effect of Peri-aortic adipose tissue on aortic diameter*								
(27)	C57BL/6J mice	Deletion of AT1a in PVAT	Ang-II	AAA	13	28 days	2.5 ± 1.8	Induction of macrophage migration by conditioned medium from angiotensin II–stimulated wild-type adipocytes was suppressed by treatment with an Osteopontin-neutralizing antibody. VAT transplantation more potently attenuated aortic aneurysm formation in OPN deficient mice than wild type
				AAA with OPN−/−	11		1.2 ± 0.7	
(28)	Sprague-Dawley rats	Peri-aortic PVAT removed	Hypo-perfusion	PVAT intact	5	28 days	NR	PVAT plays important roles in the differentiation of MSCs into adipocytes in response to vascular hypo-perfusion. The decreased number of adipocytes in the PVAT-removed vascular wall might be associated with the decreased AAA diameter.
				PVAT removed	7			
				Control	18		3.8 ± 0.5	

AAA, Abdominal aortic aneurysm; APN, Adiponectin; ADMSC, Adipose derived MSC; AdAPN, Recombinant adenoviral APN; AdGFP, Recombinant adenovirus green fluorescent protein; Ang, Angiotensin; ApoE, Apolipoprotein E; AT1a, Ang-II type 1a; BMSC, Bone marrow derived stem cells; CaCl_2_, Calcium chloride; CD, Clustered differentiation; G-CSF, Granulocyte colony stimulating factor; IL-18r, Interleukin 18 receptor; WT, Wild type; NCC, Na-Cl co-transporter; GATA, Erythroid transcription factor; HDL, High density lipoprotein; HF/HF, High-fat diet acclimatization for 20 weeks followed by high-fat diet continuation for another 8 weeks; HF/LF, High-fat diet acclimatization for 20 weeks followed by low fat diet for another 8 weeks; I.P., Intraperitoneal; INF, Interferon; IP-10, Interferon gamma-induced protein 10; LOX, Lysyl oxidase; LDL, Low density lipoprotein; LePA, Leptin antagonist; mRNA, Messenger Ribonucleic acid; MSC, Mesenchymal stem cells; MCP, Monocyte chemoattractant protein; MMP, Matrix metalloproteinase; NC, Negative control; NA, Not available; NR, Not reported; OPN, Osteopontin; ob/ob, Obese mice; PLGA, Poly lactic- co- glycolic acid; PVAT, Perivascular adipose tissue; RELMβ, Resistin-like molecule-beta; si, Small RNA inference; Th, T helper cells; TG, Triglycerides; VAT, Visceral adipose tissue; WAT, White adipose tissue; ^Aortic lumen diameters not different, however, maximal diameters were significantly different.

#### Animal Studies Investigating the Effect of Leptin in AAA

Four studies including 159 animals investigated the role of leptin in AAA pathogenesis within mouse model induced using Angiotensin-II (Ang-II) infusion (19, 21, 22) or peri-aortic calcium chloride (CaCl_2_) application (20) (**Table 1A**). These studies used mice with different backgrounds including Apolipoprotein E (ApoE) (19, 22), combined Na-Cl co-transporter and interleukin (IL)-18 receptor (20), and low density lipoprotein receptor (LDL-r) (21) deficient mice. The role of leptin in AAA pathogenesis was explored through local peri-aortic application (19, 20), intraperitoneal injection (22), and gene deficiency (20, 21).

Peri-aortic application of leptin was reported to promote a significant increase in AAA severity (diameter) in the Ang-II model (19). Leptin also promoted aortic and visceral adipose macrophage infiltration. A further study reported that perivascular implantation of white adipose tissue in obese or lean mice resulted in an increased AAA size over controls in the CaCl_2_ model (20). Perivascular implantation of adipose tissue from leptin deficient mice abolished the increase in AAA diameter size that was demonstrated when adipose tissue from leptin sufficient mice was used, suggesting a role of leptin in adipose tissue induced AAA development (20).

In contrast to the above findings, germline leptin deficiency has been reported to promote the risk of AAA rupture in obese mice infused with Ang-II but AAA size was not reported in this study (21). In keeping with a role of leptin in inhibiting AAA pathogenesis, intraperitoneal injection of leptin (600 μg/kg daily) has been reported to reduce AAA diameter and risk of rupture in the Ang-II model (22). Leptin was reported to downregulate expression of the Th2 cytokine IL-4 and mRNA levels of GATA binding protein-3 (GATA-3), a key transcription factor for Th2 polarization. In addition, leptin also upregulated Th1 cytokine interferon-gamma (INF-γ) and T-box binding transcription factor (T-bet), a key transcription factor for Th1 polarization (22). These results led the authors to suggest that leptin could be a potential treatment for the prevention of AAA formation.

**Figure 3:**
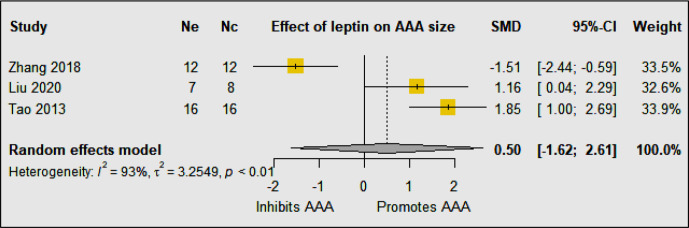
The included studies had contrasting findings most likely due to the varied methods of leptin upregulation. The meta-analysis suggested that overall upregulation of leptin had no effect on AAA diameter. Ne, Number of animals in experimental group; Nc, Number of animals in control group.

A meta-analysis of three eligible studies suggested that leptin upregulation had no significant effect on AAA diameter (SMD: 0.50 [95% CI: −1.62, 2.61]) (**Figure 3**). Overall the findings of these studies suggest that the effect of leptin depends on the method and site of up and downregulation. All four studies used the same mouse strain (C57BL/6) and experimental period but the method of leptin upregulation varied markedly which likely was the cause of the inconsistent findings. The studies reported that leptin promoted IL18 binding to inflammatory and vascular cells by upregulating the expression of IL18, the IL18-receptor, and the Na-Cl co-transporter in addition to augmenting MMP-9 synthesis (19, 20, 22).

#### Animal Studies Investigating the Effect of Adiponectin on AAA

**Figure 4:**
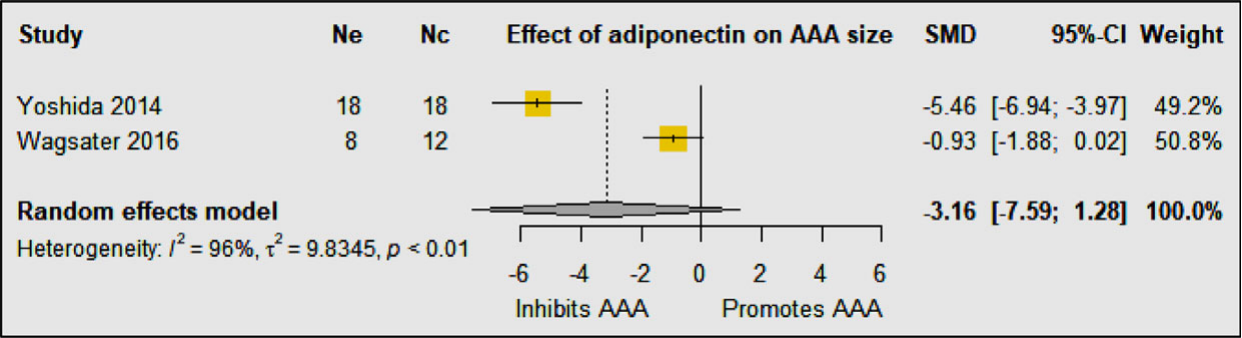
Meta-analysis suggested that upregulation of adiponectin had no significant effect on AAA diameter. Ne, Number of animals in experimental group; Nc, Number of animals in control group.

Two studies including 64 animals investigated the effect of adiponectin on AAA development in mouse models (23, 24) (**Table 1B**). In high-fat fed mice with LDL-r deficiency, intravenous injection of a recombinant adenoviral vector encoding adiponectin was reported to inhibit AAA development in the Ang-II infusion model (23). Adiponectin was reported to inhibit AAA development by suppressing aortic inflammatory cell infiltration, medial degeneration, and elastin fragmentation (23). Adiponectin was also shown to inhibit the angiotensin type-1 receptor (AT1R), downregulate expression of inflammatory cytokines, mast cell protease, and adipose inflammation, and upregulate lysyl oxidase (LOX) expression in the aortic wall (23). In keeping with this, germline deletion of adiponectin has been reported to promote AAA development in the Ang-II infusion model (24). Adiponectin-deficiency augmented the early infiltration of macrophages and increased the expression of matrix remodeling enzymes such as matrix metalloproteinase (MMP) -2 and 9 in the aortic wall. These results suggest that adiponectin protects against AAA formation through multiple different mechanisms, of which inhibiting inflammation plays a key role. Meta-analysis suggested that adiponectin upregulation did not significantly reduced AAA diameter (SMD: −3.16 [95% CI: −7.59, 1.28]) (**Figure 4**). The findings of the meta-analysis need to be considered in the context of the limited number of available studies and included animals.

#### Animal Studies Investigating the Effect of RELMβ on AAA

Two studies including 60 mice in one study and an unreported number in one study investigated the role of RELMβ in AAA development (25, 26) (**Table 1C**). Both studies used the same mouse strain (ApoE^−/−^) and experimental period. Germline deletion of RELMβ resulted in significantly reduced AAA diameter within the Ang-II infusion model (25). This was associated with reduced macrophage accumulation and decreased expression of pro-inflammatory cytokines (monocyte chemoattractant protein 1 and IL6) and MMP2 and 9 in the aortic wall. Further, it has also been reported that RELMβ expression was upregulated within the aortas of mice with Ang-II induced AAA compared to controls (26). These limited investigations suggest that RELMβ could have a role in promoting AAA formation *via* induction of pro-inflammatory cytokines. Meta-analysis was not possible due to the lack of available studies.

#### Animal Studies Investigating the Effect of PVAT on AAA

Two studies reporting the effect of PVAT in AAA development in 54 animals were identified (27, 28) (**Table 1D**). In mice deficient in osteopontin, angiotensin II type 1a (AT1a) deficiency of PVAT significantly attenuated the size of AAA developing, macrophage infiltration, and gelatinolytic activity in the Ang-II model (27). Another study investigated the effect of AAA development following the removal of PVAT in a hypo-perfusion induced AAA rat model (28). In this model, the authors intended to isolate the oxygen supply and induce hypoxia in the infra-renal aortic region. First, the PVAT was removed from around the infra-renal aorta to prevent oxygen supply *via* the adventitial vasa vasorum (VV). Following this, a polyurethane catheter was inserted *via* a small incision in the infra-renal aorta to separate the lumen blood flow from the aortic wall, thus isolating the oxygen supply to the aortic wall from the blood directly. These two steps reduced the oxygen supply and induced hypoxia in the infra-renal aortic region without affecting the systemic circulation, promoting AAA formation. It was reported that 28 days after the removal of the PVAT the number of CD44+ and CD90+ cells and adipocytes in the AAA wall was significantly less when PVAT had been removed. Mice that has PVAT removed were reported to have significantly smaller AAA diameter than controls (28). Evidence from other animal studies has suggested that PVAT contributes to intimal hyperplasia *via* leptin signaling (29). Samples from mouse models of AAA show infiltration by neutrophils, mast cells, and T-cells and expression of cathepsin K and S within PVAT which may contribute to AAA development (30). The exact role of PVAT in AAA development needs further investigation.

#### Quality of Prior Animal Studies Investigating Adipokines and PVAT in AAA

All studies reported an ethics approval statement, AAA model type and included relevant control groups (**Table 2**). None of the studies reported sample size estimates. Two studies reported that the assessors were blinded during the aortic diameter and/or adipokine assessments (20, 21). One study reported the reproducibility of aortic diameter assessment (20). Three studies did not report aortic diameter following the assessment period (21, 26, 28). One study did not report either the age or weight of the animals used in the study (26). One study did not report the number of animals included in each group (26). Overall, one (20), eight (19, 21–25, 27, 28), and one (26) studies were considered to have low, moderate, and high risk of bias respectively.

**Table 2:** Quality assessment of animal studies investigating the effect of adipokines and PVAT in AAA.

Reference	Reported the ethics approval	Reported animal strain used in each group	Reported animal age in each group	Reported the number of animals used in each group	Reported the sample size estimate	Explained the methods for AAA model development	Relevant controls included	Reported aortic diameter in both AAA and controls as Mean ± SD or SEM	Reported the methods used to assess aortic diameter	Reported the reproducibility of aortic diameter assessment	Mentioned the methods to randomize the animals between groups	Assessors were blinded during the cytokine/AAA assessment experiments	Total score	Quality score (%)
(19)	1	1	1	1	0	1	1	1	1	0	0	0	8	66.6
(20)	1	1	1	1	0	1	1	1	1	1	0	1	10	83.3
(21)	1	1	1	1	0	1	1	0	0	0	0	1	7	58.3
(22)	1	1	1	1	0	1	1	1	1	0	0	0	8	66.7
(23)	1	1	1	1	0	1	1	1	1	0	0	0	8	66.7
(24)	1	1	1	1	0	1	1	1	1	0	0	0	8	66.7
(25)	1	1	1	1	0	1	1	1	1	0	0	0	8	66.7
(26)	1	0	0	0	0	1	1	0	0	0	0	0	3	37.5
(27)	1	1	1	1	0	1	1	1	1	0	0	0	8	66.7
(28)	1	1	1	1	0	1	1	0	0	0	0	0	6	50

AAA, Abdominal aortic aneurysm; PVAT, Perivascular adipose tissue; SD, Standard deviation; SEM, Standard error of the mean; %, Percentage; Yes, 1; No, 0; Total score of 12, 100%.

#### Evidence From Human Studies For A Role Adipokines and PVAT in AAA Pathogenesis

##### Studies Investigating the Association of Adipokines With AAA Diagnosis

Five studies were identified studying the association of circulating adipokines, including DPP-4, leptin, resistin, and adiponectin, with AAA in 1146 cases compared with 837 control participants (30–34) (**Table 3**). The design of the studies included retrospective cohort, case control, and prospective cohort. Two studies matched cases and controls for age (33, 34) and sex (32, 34). Three studies reported that controls were healthy (30, 32, 34).

**Table 3:** Examples of clinical studies investigating the association of adipokines with AAA.

Reference	Study design	Groups	Sample size	Aortic diameter	Adipokine levels/HR [95% CI]^*^	p value	Confounders adjusted for analyses
*A) Studies estimating the association of DPP-4 activity and AAA*							
(32)	Case control study	Control	20	NR^$^	9.9 ± 9.2	<0.05	None
		Small AAA	16	3.6 ± 0.7^$^	19.2 ± 8.2		
		Large AAA	77	5.9 ± 1.4^$^ (cm)	30.4 ± 13.9		
*B) Studies estimating the association of leptin and AAA*							
(31)δ	Retrospective cohort study	AAA	701	NR	HR: 0.8 (0.7–1.0)	NR	Age, sex, race, smoking status, pack-years of smoking, height, hypertension, HDL, LDL, TC, and PAD
(33)	Prospective cohort study	No AAA	174	NR	7.3 (4.7–11.4)	0.19	Age, smoking, BMI, carotid artery stenosis, DM, arterial hypertension, HDL, LDL, TC, TG, CRP, and statins
		AAA	15	NR	9.7 (4.9–17.3)		
(34)δ	Cohort from population based RCT	Control	634	19–22^#^ (mm)	12.6 ± 9.9	<0.01	Age, dyslipidemia, hypertension, smoking, CHD, DM, WHR, and serum glucose
		AAA	318	≥30 (mm)	16.5 ± 16.7		
*C) Studies estimating the association of Resistin and AAA*							
(33)	Prospective cohort study	No AAA	174	NR	9.4 (7.6–12.3)	0.08	Age, smoking, BMI, carotid artery stenosis, DM, arterial hypertension, HDL, LDL, TC, TG, adiponectin, leptin, CRP, and statins
		AAA	15	NR	12.7 (8.5–16.8)		
(34)δ	Cohort from population based RCT	Control	634	19–22^#^ (mm)	20.7 ± 10.5	<0.01	Age, dyslipidemia, hypertension, smoking, CHD, DM, WHR, and serum glucose
		AAA	318	≥30 (mm)	27.6 ± 12.1		
*D) Studies estimating the association of adiponectin and AAA*							
(33)	Prospective cohort study	No AAA	174	NR	4.2 (2.7–6.4)	0.86	Age, smoking, BMI, carotid artery stenosis, DM, arterial hypertension, HDL, LDL, TC, TG, leptin, CRP, and statins
		AAA	15	NR	3.5 (2.1–11.5)		
(34)δ	Cohort from population based RCT	Control	634	19–22^#^ (mm)	9.9 ± 4.7	<0.01	Age, dyslipidemia, hypertension, smoking, CHD, DM, WHR, and serum glucose
		AAA	318	≥30 (mm)	10.8 ± 4.7		
(30)^**^	Case control study	Control	9	NR	97.5 ± 39.4	NR	None
		AAA	19	63.0 ± 12.2^$^	27.8 ± 5.4		

AAA, Abdominal aortic aneurysm; BMI, Body mass index; CHD, Coronary heart disease; CI, Confidence interval; CRP, C reactive protein; cm, Centimeter; DM, Diabetes mellitus; DPP-4, Dipeptidyl peptidase-4; HR, Hazard ratio; HDL, High density lipoprotein; LDL, Low density lipoprotein; NR, Not reported; mm, Millimeter; PAD, Peripheral artery disease; RCT, Randomized controlled trial; TC, Total cholesterol; TG, Triglycerides; WHR, Waist to hip ratio; *Adipokine levels were either mentioned as mean ± S.D. or median (25^th^–75^th^ percentile range); **Adipokine levels data extracted from graph; ᵟSub-analysis data assessed adipokines from a larger cohort; ^#^Aortic diameter measured using ultrasound (US); ^$^Aortic diameter measured using Computed tomography (CT).

A case-control study reported that plasma DPP-4 enzyme activity was significantly increased in both large and small AAA patients compared to controls and positively correlated with AAA diameter (32) (**Table 3A**). However, circulating DPP-4 plasma levels were similar between control and small AAA participants and significantly lower in people with large AAA (32). This study suggested that increased DPP-4 activity may play a role in AAA pathogenesis but larger studies are needed.

**Figure 5:**
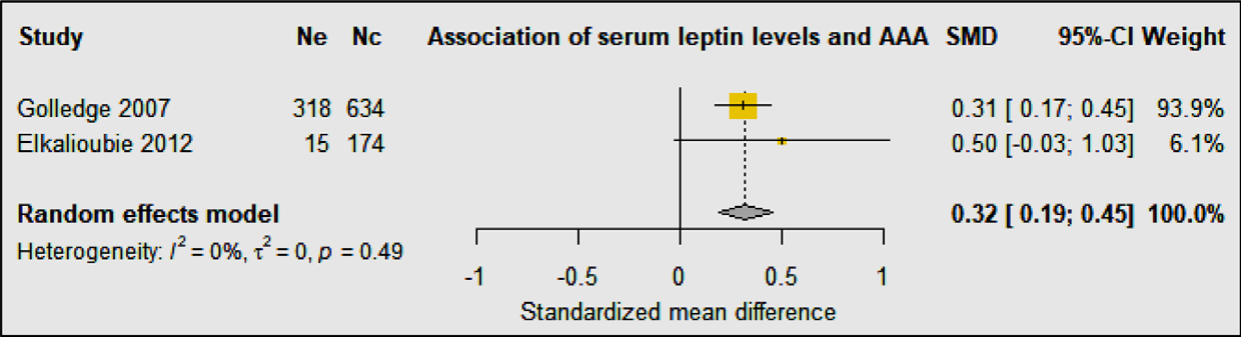
Forest plot suggesting the association of high circulating leptin concentrations with AAA diagnosis. Ne, Number of patients in experimental group; Nc, Number of patients in control group.

Three studies including 1,034 AAA patients and 808 controls investigated the association of circulating leptin concentrations with AAA (31, 33, 34) (**Table 3B**). A cohort sample of 701 patients from the Atherosclerotic Risk in Communities (ARIC) study suggested that plasma leptin levels were inversely associated with AAA diagnosis after adjusting for confounders including age, sex, race, smoking status, pack-years of smoking, height, hypertension, high and low density lipoproteins (HDL and LDL), total cholesterol, and peripheral artery disease, but the study did not report aortic diameter (31). A prospective cohort study showed that serum levels of leptin were similar in AAA and control participants after adjusting for age, smoking, body mass index (BMI), carotid artery stenosis, diabetes, hypertension, lipids, C-reactive protein (CRP), and statin prescription, suggesting that leptin was not associated with AAA (33). Another population based study reported leptin levels in 952 men screened for AAA, 318 of whom had an AAA diagnosed. Results from this study suggested that leptin levels were not independently associated with AAA after adjusting for confounders including age, smoking, BMI, carotid artery stenosis, diabetes, hypertension, HDL, LDL, total cholesterol, triglycerides, CRP, and statins (34). A meta-analysis suggested that overall circulating leptin levels were higher in people diagnosed with an AAA than controls (SMD: 0.32 [95% CI: 0.19, 0.45]) (**Figure 5**). It was not possible to adjust this meta-analysis for confounding risk factors.

**Figure 6:**
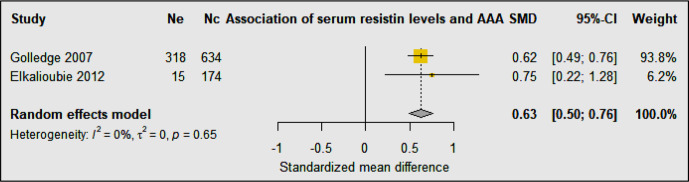
Forest plot suggesting the association of high circulating resistin concentrations with AAA diagnosis. Ne, Number of patients in experimental group; Nc, Number of patients in control group.

Two studies including 333 AAA patients and 808 controls investigated the association of serum resistin levels with AAA (33, 34) (**Table 3C**). A prospective cohort study showed a trend toward higher resistin levels in participants with AAA compared to controls (33). Multivariate analyses showed that serum resistin levels were independently associated with AAA after adjusting for age, smoking, BMI, carotid artery stenosis, diabetes, hypertension, HDL, LDL, cholesterol, triglycerides, adiponectin, leptin, CRP, and statin medication (33). A population-based study in men found that serum resistin concentrations were independently associated with AAA after adjusting for age, dyslipidemia, hypertension, smoking, CHD, and diabetes mellitus, waist-hip ratio, and serum glucose (34). Meta-analysis of these studies suggested that serum resistin levels were significantly higher in people diagnosed with AAA compared to controls (SMD: 0.63 [95% CI 0.50, 0.76]) (**Figure 6**).

**Figure 7:**
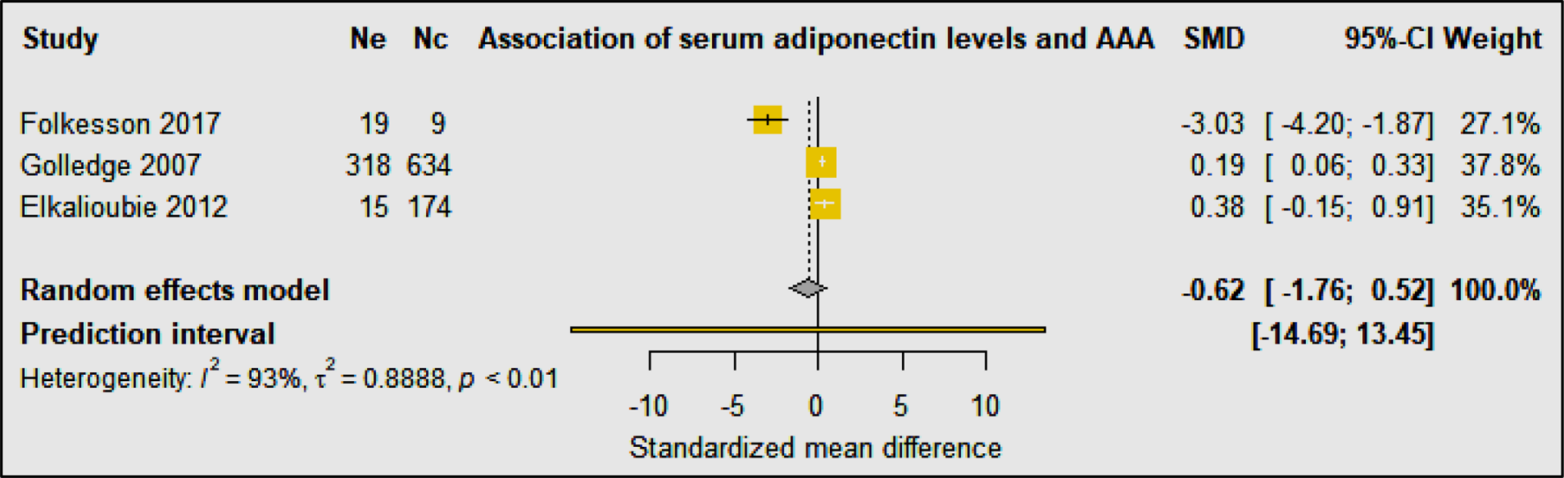
Forest plot suggesting no association of circulating adiponectin concentrations with AAA diagnosis. Ne, Number of patients in experimental group; Nc, Number of patients in control group.

Three studies including 352 AAA patients and 817 controls investigated the association of circulating adiponectin with AAA (30, 33, 34) (**Table 3D**). A prospective cohort study showed no independent association of serum levels of adiponectin with AAA after adjusting for age, smoking, BMI, carotid artery stenosis, diabetes, hypertension, lipids, CRP, and statin prescription (33). A population-based study found that serum levels of adiponectin were associated with AAA ≥30 mm, but not AAA ≥40 mm, suggesting that adiponectin may play a role in the early stages of AAA development (34). A case-control study reported that serum levels of adiponectin were significantly lower in AAA patients compared to healthy control organ donors, however, adiponectin was significantly increased in PVAT surrounding the AAA, suggesting that adipocytes surrounding the aorta may be a source of inflammatory leukocytes that are attracted by adipocytes undergoing necrosis and by pro-inflammatory ceramides (30). Meta-analysis suggested that serum adiponectin levels were not significantly associated with AAA diagnosis (SMD: −0.62 [95% CI −1.76, 0.52]) (**Figure 7**). Phenotypic changes in PVAT resulting from vascular injury have been reported to promote neointimal formation *via* release of adipocytokines which in turn regulate inflammation, vascular smooth muscle cell proliferation, endothelial dysfunction, fibroblast activation and migration and neovascularization (35). Furthermore, high fat diet or smoking have also been suggested to promote vascular inflammation, reactive oxygen species production, and matrix degradation that augments AAA formation (36).

##### Quality of Human Studies Investigating the Association of Adipokines With AAA

All studies measured adipokines in AAA cases and a comparator group (**Table 3**) (30–34). Three studies reported the results after adjusting for confounders (31, 33, 34). Three studies reported the methods used for aortic diameter imaging (30, 32, 34). Two studies reported the methods used for aortic diameter assessment (32, 34). One study imaged both AAA cases and the comparator groups (34). One study reported the sample size estimates used (31).

#### Studies Examining the Association of PVAT With AAA

**Figure 8:**
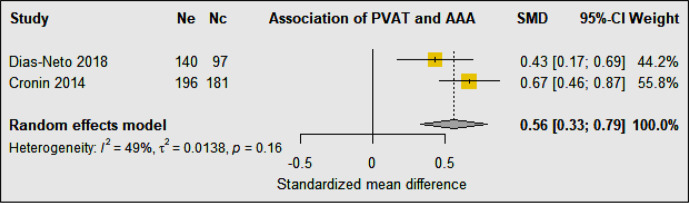
Forest plot suggesting a significant association between greater perivascular adipose tissue to abdominal adipose tissue ratio and AAA diagnosis. Ne, Number of patients in experimental group; Nc, Number of patients in control group.

Three studies were identified investigating peri-aortic and visceral adipose tissue in 3,337 AAA cases compared with 382 control participants (37–39) (**Table 4**). The design of the studies included retrospective, prospective, and longitudinal cohort and case-control studies. All three studies matched the AAA cases and comparators for age and sex (37–39). In a multicenter retrospective case-control study, comparison of participants with asymptomatic AAA, aortoiliac occlusive disease (AIOD), and healthy controls suggested that AAA was associated with higher PVAT density and the visceral to subcutaneous adipose tissue (VAT/SAT) ratio was significantly greater in AAA patients (37). This association persisted after adjustment for cardiovascular risk factors and other diseases. Another case-control study compared AAA and intermittent claudication control subjects and also reported that the visceral to total abdominal adipose volume ratio was significantly greater in participants diagnosed with AAA, but the association was abolished after adjusted for age, heart disease, diabetes, smoking, sex, and hypertension (38). In a Framingham study including 3,001 individuals, the authors reported a significant association between peri-aortic fat deposition and larger aortic dimensions after adjusting for age, sex, cardiometabolic risk factors, and BMI, further supporting the notion that fat depots present around the aorta may contribute to its remodeling (39). Meta-analysis suggested that PVAT to abdominal adipose tissue ratio was significantly associated with AAA diagnosis (SMD: 0.56 [95% CI 0.33, 0.79]) (**Figure 8**).

**Table 4:** Examples of clinical studies investigating the association of perivascular adipose tissue with AAA.

Reference	Study design	Groups	Sample size	Aortic diameter	PVAT to AAT ratio	p value	Confounders adjusted for analyses
(37)	Retrospective case control study	Control	97	2.0 ± 0.2^$^ (cm)	1.2 ± 0.7^*^	0.006	Gender, diabetes, hypertension, smoking, CHD, PAD, BMI, anticoagulation, antiplatelet therapy, vasodilator, diuretics, CCB, BB, statins
		AIOD	104	2.1 ± 0.4^$^ (cm)	1.2 ± 0.8^*^		
		aAAA	140	6.1 ± 1.4^$^ (cm)	1.5 ± 0.7^*^		
(38)	Case control study	AAA	196	50.0 (42.0–57.0)^$^ (mm)	0.5 (0.4, 0.6)^**^	0.007	Age, CHD, diabetes, ever smoked, sex and hypertension
		IC	181	21.0 (20.0–24.0)^$^ (mm)	0.4 (0.3, 0.5)^**^		
(39)^δ^	Framingham Heart - Longitudinal cohort study	AAA in women	1,474	17 ± 2^$^ (mm)	HR (95% CI): VAT - 0.3 (0.2 to 0.4)δ AAT - 0.3 (0.2 to 0.3)δ	<0.01	Age, sex, cardiometabolic risk factors, and BMI
		AAA in men	1,527	19 ± 2^$^ (mm)			

AAA, Abdominal aortic aneurysm; aAAA, Asymptomatic AAA; AAT, Abdominal peri-aortic fat; AIOD, Aortoiliac occlusive disease; BB, Beta blockers; BMI, Body mass index; CCB, Calcium channel blockers; CHD, Coronary heart disease; CI, Confidence interval; cm, Centimeter; HR, Hazard ratio; IC, Intermittent claudication; mm, Millimeter; PAD, Peripheral artery disease; PVAT, Perivascular adipose tissue; VAT/SAT, Visceral adipose tissue/subcutaneous adipose tissue; *Data reported as mean ± Standard deviation; **Data reported as median with 25^th^ and 75^th^ percentile range; ᵟ, Data reported as hazard ratio with 95% confidence interval; ^$^Aortic diameter measured using Computed tomography (CT).

#### Quality of Human Studies Investigating the Association of PVAT With AAA

All studies reported the methods used to image the aorta, methods used for aortic diameter assessment and adjusted for potential confounders during analysis (**Table 4**) (37–39). Two studies reported the sample size estimate used (37, 38). Only one study blinded the observer during analysis of experimental data (37).

#### Overall Quality Assessment of All Included Human Studies

Overall, two (37, 38), two (34, 39), and four (30–33) of the clinical research studies were considered to have low, moderate, and high risk of bias respectively (**Table 5**).

**Table 5:** Quality assessment of clinical studies investigating the role of adipokines and PVAT in AAA.

Reference	Sample size estimate reported	Age matched controls	Sex matched controls	Controls and AAA cases imaged	Method of aortic diameter imaging	Method of AAA diameter assessment	Analysis by blinded observer	Confounders were adjusted for analyses	Total	Quality score (%)
(31)	1	0	0	0	0	0	0	1	2	25
(32)	0	0	1	0	1	1	0	0	3	37.5
(33)	0	1	0	0	0	0	0	1	2	25
(34)	0	1	1	1	1	1	0	1	6	75
(30)	0	0	0	0	1	0	0	0	1	12.5
(37)	1	1	1	1	1	1	1	1	8	100
(38)	1	1	1	1	1	1	0	1	7	87.5
(39)	0	1	1	0	1	1	0	1	5	62.5

AAA, Abdominal aortic aneurysm; PVAT, Perivascular adipose tissue; %, Percentage; Yes = 1; No = 0; Score of 8 = 100%.

## Conclusions

Overall this systematic review suggests inconsistent effects of adipokines and PVAT on AAA development within rodent AAA models. Circulating levels of leptin and resistin and PVAT to abdominal adipose tissue ratio, but not adiponectin, were found to be significantly greater in people diagnosed with AAA compared to controls. Given the limited number of eligible animal and human studies, more evidence is needed before any robust conclusions about the role of adipokines and PVAT in AAA pathogenesis can be made. The quality assessments suggested that this past research had a moderate risk of bias. 

## Data Availability Statement

The original contributions presented in the study are included in the article/supplementary material. Further inquiries can be directed to the corresponding author.

## Author Contributions

JG conceptualized the study. ST performed the screening of studies, data extraction, and analysis. Both ST and JG contributed to preparation of the manuscript. JG critically assessed the manuscript. All authors contributed to the article and approved the submitted version.

## Funding

Funding from the National Health and Medical Research Council (1063476 and 1022752), James Cook University, The Townsville Hospital and Health Services Study, Education and Research Trust Fund, and Queensland Government supported this work. Jonathan Golledge holds a Practitioner Fellowship from the National Health and Medical Research Council (1117061) and a Senior Clinical Research Fellowship from the Queensland Government, Australia.

## Conflict of Interest

The authors declare that the research was conducted in the absence of any commercial or financial relationships that could be construed as a potential conflict of interest.
